# Cell Factory Engineering of Undomesticated *Bacillus* Strains Using a Modified Integrative and Conjugative Element for Efficient Plasmid Delivery

**DOI:** 10.3389/fmicb.2022.802040

**Published:** 2022-04-26

**Authors:** Da-Eun Jeong, Man Su Kim, Ha-Rim Kim, Soo-Keun Choi

**Affiliations:** ^1^Infectious Disease Research Center, Korea Research Institute of Bioscience and Biotechnology (KRIBB), Daejeon, South Korea; ^2^Department of Biosystems and Bioengineering, KRIBB School of Biotechnology, University of Science and Technology (UST), Daejeon, South Korea

**Keywords:** *Bacillus*, integrative and conjugative element, transformation, cell factory, cytosine base editor, undomesticated strain

## Abstract

A large number of *Bacillus* strains have been isolated from various environments and many of them have great potential as cell factories. However, they have been rarely developed as cell factories due to their poor transformation efficiency. In this study, we developed a highly efficient plasmid delivery system for undomesticated *Bacillus* strains using a modified integrative and conjugative element (MICE), which was designed to be activated by an inducer, prevent self-transfer, and deliver desired plasmids to the recipient cells. The MICE system was demonstrated to successfully introduce a *gfp*-containing plasmid into all 41 undomesticated *Bacillus subtilis* strains tested and eight other *Bacillus* species. The MICE was used to deliver a cytosine base editor (CBE)-based multiplex genome-editing tool for the cell factory engineering of the *Bacillus* species. The introduced CBE enabled one-step inactivation of the major extracellular protease genes of the tested strains. The engineered strains were used as hosts for heterologous expression of nattokinase, which resulted in various enzyme expression levels. The results suggested that the MICE and CBE systems can be powerful tools for genetic engineering of undomesticated *Bacillus* strains, and greatly contribute to the expansion of the *Bacillus* cell factory.

## Introduction

*Bacillus* species are among the most important industrial microorganisms that have been widely used for a variety of applications like the production of various enzymes and metabolites, as agricultural agents, in traditional food fermentations, and as hosts for heterologous protein expression (Caulier et al., 2019; Su et al., 2020). The advantages of *Bacillus* as a protein-production host are that it can secrete large amounts of protein from the cells, mass culture is well established, and most of the *Bacillus* strains are safe for humans (Westers et al., 2004). There are recent reports showing the potential of *Bacillus* strains for medical use. Fengycin, produced by *Bacillus* species eliminates the pathogen *Staphylococcus aureus* from the human body (Piewngam et al., 2018). Many health benefits as probiotics of *Bacillus* species such as anti-oxidant, anti-cancer, anti-diabetic and immuno-modulatory effects have been documented (Elshaghabee et al., 2017).

The genus *Bacillus* consists of more than 280 species (Gupta et al., 2020). The domesticated *Bacillus subtilis* strain 168 has been largely handled as a cell factory because its physiology and genetics are extensively studied. Reportedly, undomesticated ATCC 6051 strain has a greater potential as alternative industrial host compared to strain 168, as it doesn’t exhibit auxotrophy and can reach higher cell densities on complex media (Kabisch et al., 2013). Studies using not only *B. subtilis* but also *Bacillus licheniformis* (Song et al., 2021), *Bacillus megaterium* (Vary et al., 2007), and *Bacillus pumilus* (Küppers et al., 2014) as industrial protein production hosts have been reported. The promising properties related to the production of specific enzymes and other secondary metabolites have been documented in less-characterized strains such as *Bacillus sonorensis*, *Bacillus mycoides*, *Bacillus pseudomycoides*, *Bacillus vallismortis*, and *Bacillus mojavensis* (Heinze et al., 2018). In addition, certain *Bacillus* strains exhibit characteristics such as high resistance to toxic substrates or organic solvents (Kataoka et al., 2011; Mahipant et al., 2019), or can grow over extended ranges of salinity (Ibarra-Villarreal et al., 2021), pH (Newcombe et al., 2009) or temperature (Choonut et al., 2020), highlighting the potential of undomesticated *Bacillus* strains.

Undomesticated strains often need to be engineered to meet industrial demands. But, poor transformation efficiency has greatly limited their engineering. Transformation of *Bacillus* strains is usually performed through natural competence, electroporation, or conjugation. Natural competence method is commonly used in *B. subtilis* and depends on the expression of the competence machinery genes (Dubnau and Blokesch, 2019). Electroporation is also frequently used in various *Bacillus* strains (Zhang et al., 2015). Transformation of undomesticated *Bacillus* strains using these two methods is frequently inefficient or even impossible, while conjugation methods often solve the problem due to their broad-host-range nature. The well-known conjugal DNA transfer of *Bacillus* is assisted by a complex machinery for DNA translocation encoded in the *Escherichia coli* S17-1 chromosome (Simon et al., 1983) or *B. subtilis* natto plasmid pLS20 (Koehler and Thorne, 1987). However, many undomesticated strains are difficult to transform even with conjugation. Recently, it was reported that DNA was efficiently transferred to various undomesticated bacteria using engineered chromosomal integrative and conjugative elements (ICE) of *B. subtilis* (Brophy et al., 2018). However, the engineered system is difficult to use for marker-free genome engineering or serial editing of multiple genes in recipient cells, because it integrates the transferred DNAs with a selectable marker into a specific site of the recipient genome. Therefore, an effective plasmid delivery system is required in the marker-free engineering of undomesticated *Bacillus* strains for cell factory development.

*Bacillus* genome engineering for industrial applications has been carried out in a marker-free manner *via* counter-selectable markers, such as *upp* (Fabret et al., 2002), *araR* (Liu et al., 2008), *mazF* (Zhang et al., 2006), and a synthetic gene circuit (Jeong D. E. et al., 2015). However, recent editing tools are focused on a clustered regularly interspaced short palindromic repeat (CRISPR)-Cas9 and its derivatives. The sequence-specific target programming capacity of Cas9 has made it a powerful and indispensable genome-editing tool (Tyagi et al., 2020). In *B. subtilis*, several highly efficient genome-editing protocols using the CRISPR-Cas9 (Altenbuchner, 2016; Westbrook et al., 2016; So et al., 2017), and a subsequent serial genome-editing method for multiple targets have been reported (Lim and Choi, 2019). Other systems, such as CRISPR/Cpf1 assisted multiple-genes editing (Wu et al., 2020), CRISPRi-guided multiplexed fine-tuning of metabolic flux (Dong et al., 2020), and CRISPR/Cas9n-based multiplex genome editing (Liu et al., 2019) have also been documented. In addition, a tool called cytosine base editor (CBE), in which a nuclease-deactivated Cas9 (dCas9) is fused with a cytidine deaminase, has been developed for multiplex genome editing in *B. subtilis* (Kim et al., 2021). Most CRISPR-based genome-editing tools can be applied to undomesticated strains if an efficient plasmid delivery system is established.

In this study, an efficient plasmid transfer system was developed using a modified integrative and conjugative element (MICE) and applied to deliver a CBE-based multiplex genome editing tool for cell factory engineering of undomesticated *Bacillus* strains. We believe that the MICE system may be a powerful tool for the development of undomesticated *Bacillus* strains as cell factories.

## Materials and Methods

### Strains and Culture Condition

All recipient and donor strains used in this study are listed in Supplementary Tables 1, 2. *Escherichia coli* MC1061 and DH5α were used to construct recombinant plasmids. *E. coli* cells were cultured in LB (Difco, Detroit, MI, United States) medium or agar at 37°C. *Bacillus* donor strains for *Bacillus*-to-*Bacillus* conjugation (BBC) and MICE were grown in LB medium at 37°C. The growth conditions of recipient strains are indicated in Supplementary Table 1. When required, the medium was supplemented with ampicillin (100 μg/ml), chloramphenicol (15 μg/ml for *Bacillus atrophaeus* and *B. pumilus*, 50 μg/ml for *B. licheniformis*, and 5 μg/ml for other *Bacillus* strains), tetracycline (10 μg/ml), spectinomycin (100 μg/ml), polymyxin B (10 μg/ml), D-xylose (1%, w/v), D-alanine (100 μg/ml) (Sigma), 1 mM IPTG (Isopropyl β-D-thiogalactopyranoside), and skim milk (1%, w/v).

### Donor Construction

The plasmids and primers used in this study are listed in Supplementary Tables 3, 4, respectively. All-in-one CRISPR plasmid (pACas9T) used for donor construction was constructed as follows. To construct a pHPCas9spac, *Bam*HI- and *Sma*I-digested pHPspac (Kim et al., 2014) was ligated with the *Bam*HI- and *Sma*I-digested fragment amplified by PCR from pHCas9 (So et al., 2017) using primers Cas9F and Cas9R. Then, the *rep*-*cat* cassette and *oriT*_*e*_ fragment [*oriT* for *E. coli*-to-*Bacillus* conjugation (EBC)] were PCR amplified using primers pADallF/pADallR and oriTF/oriTR from pAD123 (Dunn and Handelsman, 1999) and pHCas9, respectively, and were fused with *Xho*I- and *Nar*I-digested pHCas9spac using the Cold Fusion Cloning Kit (System Biosciences Inc., Palo Alto, CA, United States). The resulting plasmid was digested with *Xho*I and *Bsr*GI, and then fused with the sgRNA cassette amplified from pAgR (So et al., 2017) using primers gRNAF and gRNAR to construct pACas9T plasmid. The oligonucleotides containing the 20-bp gRNA generated by mixing synthetic primers (Supplementary Table 4) were ligated with the *Aar*I -digested pACas9T plasmid. The sgRNA synthesis was under the control of P*_*ara*_* promoter (So et al., 2017). For cloning of the donor DNA cassette amplified by fusion PCR using primers listed in Supplementary Table 4, pACas9T containing targeting gRNA was digested with *Spe*I and *Xma*I, and the large fragment was fused to the donor DNA cassette using a cold fusion cloning kit to produce the following CRISPR plasmids: pACas9T-dalrA, pACas9T-dnicK, and pACas9T-dsigK (Supplementary Table 3). For CRISPR-Cas9 editing, the CRISPR plasmid-containing cells were spread on an LB agar plate supplemented with 1 mM IPTG and chloramphenicol (5 μg/ml). Resulting colonies were screened by colony PCR to confirm the mutation. The plasmid curing was performed as described previously (So et al., 2017).

To construct the BBC donor, *B. subtilis* BS5418 (*B. subtilis* BSK1 *thrC*::P*_*xyl*_*-*comK*) was constructed by transferring the BS5417 chromosome containing *thrC*::P*_*xyl*_*-*comK* (Jeong D. E. et al., 2015) to *B. subtilis* BSK1 (Choi et al., 2009). The transfer of plasmid pLS20 from *B. subtilis* natto KCCM 12512 to BS5418 was performed as follows: a plasmid pBC16 (Palva et al., 1990) was introduced into *B. subtilis* natto KCCM 12512 by electroporation as previously described (Meddeb-Mouelhi et al., 2012) to construct a strain BS5879 containing both pLS20 and pBC16. We used strain BS5879 as a donor cell for co-transferring the plasmids pBC16 and pLS20 to the recipient strain BS5418. The pLS20-mediated conjugation was performed as described in the section “Transformation” below. The introduction of both pLS20 and pBC16 into BS5418 was confirmed by colony PCR. To retain only pLS20 in BS5418, plasmid pBC16 was eliminated by introducing pACas9T-gTc with *tet* gene-targeting gRNA. After confirming the removal of pBC16 by colony PCR, curing of pACas9T-gTc was performed to obtain the strain BS5887. A previous report showed that deletion of alanine racemase-encoding gene *alrA* in *B. subtilis* resulted in D-alanine auxotrophy that could be used as a counter-selectable marker against donor growth (Brophy et al., 2018). To construct the *alrA* deletion mutant, pACas9T-dalrA was introduced into BS5887, and the resulting colonies were screened by colony PCR to confirm the deletion of the *alrA* gene. After curing the plasmid pACas9T-dalrA, the resulting strain BS5892 was used as a donor strain in the BBC experiment.

Modified integrative and conjugative element donor contain deletion from *rapI* to *attR*, insertion of *rapI* gene into *alrA* gene, and mutation of *oriT* in the *nicK* gene. For the deletion from *rapI* to *attR*, two DNA fragments corresponding to upstream and downstream regions of *rapI*–*attR* were amplified from the chromosome of *B. subtilis* 168 using primers yddJ-H3F/yddJ-hygR and yddN-hygF/yddN-EcoR, respectively. The hygromycin-resistance gene (*hygR*) was amplified from the chromosome of *B. subtilis* WB800N (Jeong H. et al., 2018) using primers hyg-F and hyg-R, and fused to the upstream and downstream fragments of *rapI*–*attR* by fusion PCR to obtain *rapI*::*hygR* cassette. Plasmid pUC18 was digested with *Hin*dIII and *Eco*RI, and the large fragment was fused to the *rapI*::*hygR* cassette using a cold fusion cloning kit to construct plasmid pUC-rapI::hyg. The resulting plasmid was introduced into *B. subtilis* 168 to generate strain BS5889. Next, pGEM-rapI was constructed as follows to generate a mutation in *alrA* gene for inducible *rapI* expression. Three DNA fragments corresponding to upstream and downstream regions of *alrA*, and structural gene of *rapI* were amplified from the chromosome of *B. subtilis* 168 using primers alr_front_F/alr_front_R, alr_back_F/alr_back_R, and rapI-F/rapI-R, respectively. The spectinomycin-resistance gene (*spcR*) and inducible promoter P*_*xylA*_* was amplified from the pDG1728 (Guérout-Fleury et al., 1996) and pAX01 (Härtl et al., 2001) using spcF/spcR and xyl-F/xyl-R, respectively. All fragments were fused by fusion PCR to obtain *alrA*::*spcR*-P*_*xyl*_*-*rapI* cassette. pGEM-T Easy (Promega Co., Madison, WI, United States) was digested with *Nco*I and *Pst*I, and the large fragment was fused to the *alrA*::*spcR*-P*_*xyl*_*-*rapI* cassette using a cold fusion cloning kit to construct plasmid pGEM-rapI. The resulting plasmid was introduced into BS5889 to generate strain BS5890. Finally, to eliminate the *oriT*_*ICE*_ (ICE*Bs1* origin of transfer) nicking site located within the *nicK* open reading frame (ORF), we changed nucleotide sequences of the *oriT*_*ICE*_ without changing the amino acid sequence of the NicK. Plasmid pACas9T-dnicK for *oriT*_*ICE*_ mutation was introduced into the BS5890. After confirming the *oriT*_*ICE*_ mutation by sequencing and subsequent curing of the plasmid pACas9T-dnicK, the resulting strain BS5899 was used as a major donor strain in MICE experiments. MICE donor was further mutated in the *sigK* gene by introducing plasmid pACas9T-dsigK into BS5899. The resulting strain BS5918 is a sporulation-negative mutant, which can help the screening of transconjugants during conjugation.

### Construction of Mobilizable Plasmids

The mobilizable plasmids pA3D-gfpTe, pA3D-gfpT20, and pA3D-gfpTi for expressing GFP under the control of *cry3Aa* promoter were constructed as follows. The *cry3Aa* promoter was amplified from the plasmid pMar3g (Jeong D. E. et al., 2018) using primers 3Aag-F2 and stabR2. The *gfp* gene was amplified from plasmid pAD123 using primers gfp-F1 and gfp-R1, and fused to the *cry3Aa* promoter by fusion PCR to obtain the P*_*cry*3*Aa*_*-*gfp* cassette. The PCR product containing the P*_*cry*3*Aa*_*-*gfp* cassette was digested with *Eco*RI and *Bsi*WI and inserted into corresponding sites of plasmid pDG1661 (Guérout-Fleury et al., 1996) to construct the pD3D-gfp. The plasmid pD3D-gfp was digested with *Eco*RI and *Nsi*I, and the small fragment was ligated with *Eco*RI- and *Nsi*I-digested plasmid pAD123 to construct a pA3D-gfp. For EBC, the *oriT*_*e*_ region was amplified from plasmid pAgR using primers oriTe-AatF and oriTe-SacR. Plasmid pA3D-gfp was digested with *Aat*II and *Sac*I, and the large fragment was fused to the *oriT*_*e*_ using a cold fusion cloning kit to construct pA3D-gfpTe. For BBC, the *oriT*_20_ (pLS20 *oriT*) region was amplified by colony PCR using primers oriT20-BamF and oriT20-XbaR from *B. subtilis* natto KCCM 12512. Plasmid pA3D-gfpTe was digested with *Bam*HI and *Xba*I, and the large fragment was fused to the *oriT*_20_ using a cold fusion cloning kit to construct pA3D-gfpT20. For MICE, the *oriT*_*ICE*_ (ICE*Bs1 oriT*) region was amplified by PCR using primers oriTICE-BamF and oriTICE-XbaR from the chromosome of *B. subtilis* 168. Plasmid pA3D-gfpTe was digested with *Bam*HI and *Xba*I, and the large fragment was fused to the *oriT*_*ICE*_ using a cold fusion cloning kit to construct pA3D-gfpTi. The mobilizable plasmid pm3D-aprNTi for the expression of a nattokinase gene *aprN* under the control of *cry3Aa* promoter was constructed as follows. The plasmid pA3D-gfpTi was digested with *Aat*II and *Nsi*I, and the small fragment was ligated with *Aat*II- and *Nsi*I-digested plasmid pMGoldi-sCBE4 to construct pm3D-gfpTi. The *cry3Aa* promoter was obtained by PCR from pA3D-gfpTi using primers P3D-sacF and stabR2. The *aprN* structural gene was amplified from the chromosome of *B. subtilis* natto KCCM 12027 using primers aprE-F2 and aprE-BWR, and fused to the *cry3Aa* promoter by fusion PCR to obtain the P*_*cry*3*Aa*_*-*aprN* cassette. Plasmid pm3D-gfpTi was digested with *Sac*I and *Bsi*WI, and the large fragment was fused to the P*_*cry*3*Aa*_*-*aprN* cassette using a cold fusion cloning kit to construct pm3D-aprNTi.

### Transformation

Transformation of *B. subtilis* through natural competence (Lim and Choi, 2019) and electroporation (Meddeb-Mouelhi et al., 2012) were carried out by previously described methods. EBC was performed as described previously with the following modifications (Teng et al., 1998). The *E. coli* S17-1 donor and *B. subtilis* recipient cells were grown at 37°C for 16 h in LB broth with appropriate antibiotics. The cultures were diluted 100-fold in fresh LB broth and further incubated until OD_600_ reached 0.6–0.8. Then, 1 ml of donor and recipient cells were harvested by centrifugation (10,000 × *g*, 3 min), washed once in 1 ml phosphate buffered saline (PBS, pH 7.2, Enzynomics, Daejeon, South Korea), and resuspended in 20 μl of LB broth. Then, 20 μl each of donor and recipient cell suspensions were mixed and spotted onto nitrocellulose membrane (0.45 μm, Advantec, Tokyo, Japan) placed on LB agar. After mating for 24 h at 37°C, the filter was transferred to a 5-ml centrifuge tube containing 1 ml of LB broth and vortexed for 1 min to remove the bacteria from the filter. The resulting suspension was serially diluted and plated on LB agar containing polymyxin B and chloramphenicol for selection of transconjugants. All the plates were incubated for 24 h at 37°C. BBC was performed as described previously with the following modifications (Koehler and Thorne, 1987). Donor and recipient strains were grown for 16 h in appropriate media and cultivation conditions (Supplementary Table 1). The cultures were diluted 20-fold with the same medium and cultured for 5 h. Then, 0.1 ml each of donor and recipient cell cultures were mixed, and 50 μl of the mixtures were spotted onto nitrocellulose membrane placed on LB agar containing 100 μg/ml D-alanine. After mating for 16 h at 37°C, the filter was transferred to a 5-ml centrifuge tube containing 1 ml PBS and vortexed for 1 min to remove the bacteria from the filter. The resulting suspension was serially diluted and plated on LB agar plate containing appropriate antibiotics for selection of transconjugants. All the plates were incubated for 24 h at 37°C. MICE-based conjugation was performed as described previously with the following modifications (Brophy et al., 2018). Donor and recipient strains were grown for 16 h in appropriate media and cultivation conditions (Supplementary Table 1). The cultures were diluted 100-fold with the same medium and further cultured until OD_600_ reached 1. During growth, expression of *rapI* was induced by adding 1% xylose to the cultures at OD_600_ = 0.2. After mixing 3 ml each of donor and recipient cultures, the mixture was compressed using a syringe onto a 0.45-μm nitrocellulose filter and the filter was placed on LB agar or TSA (tryptic soy agar, Difco, Detroit, MI, United States) containing 100 μg/ml D-alanine. After mating for 3 h (5 h for *B. megaterium* and 24 h for *B. vallismortis*) at 30°C or 37°C (refer to Supplementary Table 1), the filter was transferred to a 5-ml centrifuge tube containing 1 ml PBS and vortexed for 1 min to remove the bacteria from the filter. The resulting suspension was serially diluted and plated on LB agar or TSA plate containing appropriate antibiotics for selection of transconjugants. All the plates were incubated for 24 h at 30°C or 37°C. When the *sigK* mutant BS5918 was used as the donor strain, the mating filters were placed on LB agar containing D-alanine and incubated for 7 days at 30°C to ensure complete sporulation of the recipient. After suspending the filter with PBS, the suspension was treated at 80°C for 60 min to remove donor cells and plated on LB agar containing appropriate antibiotics for selection of transconjugants.

### Cytosine Base Editor-Mediated Multiplex Genome Editing

For CBE mutagenesis, we replaced *oriT*_*e*_ of pMGold-sCBE4 (Kim et al., 2021) with *oriT*_*ICE*_. The oriT_*ICE*_ was amplified by PCR using primers ICE-oriT-F and ICE-oriT-R from the chromosome of *B. subtilis* 168. Plasmid pMGold-sCBE4 was digested with *Hin*dIII and *Sac*I, and the large fragment was fused to the *oriT*_*ICE*_ using a cold fusion cloning kit to construct pMGoldi-sCBE4. To construct pMGoldi-xCBE4 expressing CBE under the P*_*xylA*_* promoter, P*_*xylA*_* from *B. megaterium* was amplified from pAX01 using primers xylR-xyl-F and xylR-xyl-R. Plasmid pMGoldi-sCBE4 was digested with *Sac*I and *Fse*I, and the large fragment was fused to the P*_*xylA*_* using a cold fusion cloning kit to construct pMGoldi-xCBE4. The gRNAs used for multiplex genome editing (Supplementary Table 4) were designed and cloned using Golden-Gate assembly as previously reported (Kim et al., 2021). The CBE was under the inducible promoters P*_*spac*_* or P*_*xylA*_*, but a leaky expression without IPTG or xylose inducers was sufficient to induce the mutations. Transconjugants were analyzed by DNA sequencing to confirm the mutations. The plasmid curing of the identified mutants were performed and then used in further experiments.

### Analytical Methods

For the measurement of GFP fluorescence, cells were grown for 16 h in tryptic soy broth (TSB; Difco, Detroit, MI, United States) medium. The culture was diluted 100-fold in fresh TSB medium and cultured until they reached late stationary phase (18 h after entering stationary phase). The GFP fluorescence of the cultured samples were measured with a TriStar2 microplate reader (Berthold Technologies, Bad Wildbad, Germany) as previously reported (Jeong D. E. et al., 2018). To determine protease activity, the supernatants of cultured samples were measured using azocasein as a substrate according to a previously described method (Jeong D. E. et al., 2018). All measurements were carried out at least three times. All data generated or analyzed in this study are included in the published article.

## Results

### Transformation of Undomesticated *B. subtilis* Strains

**FIGURE 1:**
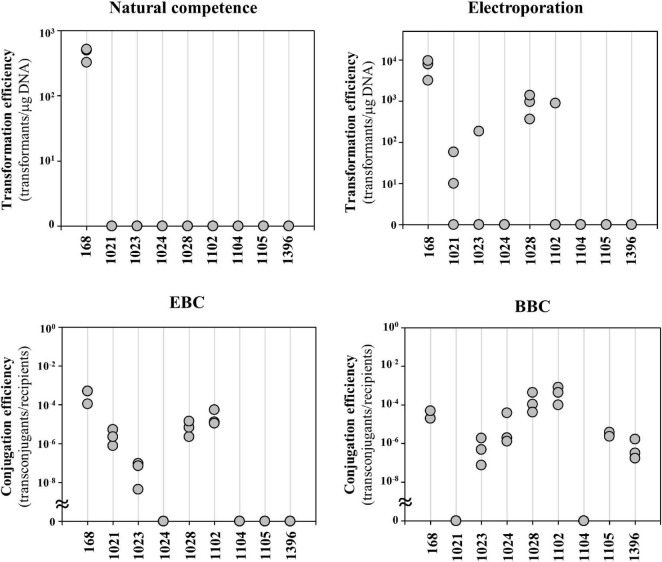
Comparison of conventional transformation and conjugation methods for undomesticated *Bacillus subtilis* strains. Transformation efficiency for natural competence and electroporation was calculated as colony forming unit per μg DNA used. *E. coli*-to-*Bacillus* conjugation (EBC) used *E. coli* S17-1 as a donor. *Bacillus*-to-*Bacillus* conjugation (BBC) used *B. subtilis* 168 containing plasmid pLS20 as a donor. The conjugation efficiency for EBC and BBC were calculated by dividing the number of transconjugants by the number of recipient cells. The numbers are KCTC strain numbers and the 168 indicates *B. subtilis* strain 168. Three independent experiments were performed and the results are presented as closed circles.

Engineering of undomesticated *Bacillus* strains is challenging due to poor transformation efficiency. Here, we compared several previously known transformation and conjugation methods on eight undomesticated *B. subtilis* strains obtained from Korean Collection for Type Cultures (KCTC) (Figure 1 and Supplementary Table 1). The results showed that the natural competence method transformed only the domesticated strain 168. Electroporation transformed four strains, 1021, 1023, 1028, and 1102. The 1021 strain was transformed twice in three independent trials, while the strains 1023 and 1102 were transformed only in one out of three. The other four strains were untransformed by both methods. Because conjugation sometimes solves the problem, it was introduced for transformation of undomesticated strains. In the EBC method, *E. coli* S17-1 strain carrying the transfer genes of IncP–type plasmid RP4 in the chromosomes was used as a donor (Simon et al., 1983). The EBC method was able to transform only the same four strains as electroporation. BBC used a *B. subtilis* 168 containing a plasmid pLS20 as a donor. The pLS20, a 65-kb-conjugative plasmid derived from *B. subtilis* natto, is capable of transferring plasmids containing a mobile origin (*oriT*) to recipient cells (Koehler and Thorne, 1987). The BBC method is more efficient than the other methods; but, strains 1021 and 1104 were still untransformed. Therefore, a broad-host-range method for difficult-to-transform strains is needed.

### Modification of *B. subtilis* Integrative and Conjugative Element

**FIGURE 2:**
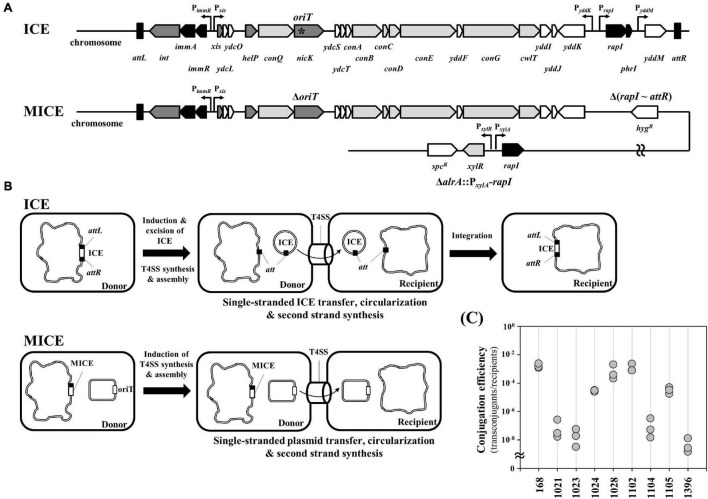
Modification of an integrative and conjugative element of *B. subtilis* to deliver plasmid. **(A)** Comparison of genetic organization between ICE and MICE. The asterisk means the oriT nicking site. **(B)** Scheme of DNA transfer mechanism through ICE or MICE. In ICE system, the chromosomal ICE of donor strain is excised using *attL* and *attR*. After transferring, the ICE is integrated into the genome of the recipient. MICE system can transfer *oriT*-carrying plasmid from donor to recipient. **(C)** Conjugation efficiency for undomesticated *B. subtilis* strains using MICE system. The conjugation efficiency was calculated by dividing the number of transconjugants by the number of recipient cells. The numbers are KCTC strain numbers and the 168 indicates *B. subtilis* strain 168. Three independent experiments were performed and the results are presented as closed circles.

Integrative and conjugative elements is a modular mobile genetic element located on the host chromosome and is an important tool of horizontal gene transfer (Figure 2A; Auchtung et al., 2016). The expression of ICE genes induces production of the type IV secretion machinery for conjugation, excision of the ICE from the chromosome, and transfer of the excised ICE to the recipient cells. In the recipient cell, the transferred single-stranded ICE undergoes circularization, second-strand synthesis, and integration into the genome (Figure 2B). Here, we modified the ICE*Bs1* to deliver desired plasmids into undomesticated *Bacillus* strains. The MICE comprises (1) deletion of *oriT* from the *nicK* gene, (2) deletion from *rapI* to *attR*, and (3) insertion of *B. megaterium xylA* promoter-regulated *rapI* gene into *alrA* gene (Figure 2A and Supplementary Table 2). In the ICE*Bs1*, the NicK DNA relaxase binds to the *oriT* of the excised circular ICE*Bs1* and makes a nick for rolling circle replication (Johnson and Grossman, 2015). In the MICE system, the chromosomal *oriT* was removed to prevent relaxase-mediated nicking of the chromosome. This enables MICE to inhibit self-transfer and assist only target plasmid transfer. As the *oriT* is located in the *nicK* gene, the deletion of *oriT* was accomplished using the CRISPR-Cas9 system by mutation of the *oriT* sequence without changing amino acid sequence of the NicK. The *attR* deletion in MICE inhibits its excision from the chromosome. Expression of ICE*Bs1* genes for conjugation is repressed by the ImmR regulator, ImmR is regulated by the protease ImmA, which in turn is activated by RapI. At low cell densities, the AbrB transition state regulator binds to the *rapI* promoter and represses its transcription, eventually inhibiting the expression of ICE*Bs1* genes. High cell density at the stationary phase inhibits *abrB* expression, which induces *rapI* expression. The expressed RapI activates ImmA, which cleaves the ImmR repressor, thereby inducing the expression of ICE*Bs1* genes. When the secreted PhrI accumulates in the medium, it re-enters the cell and inhibits RapI activity (Auchtung et al., 2016). Thus, removal of the *rapI* and *phrI* from the chromosome in the MICE system separates the expression of the MICE genes from the regulatory pathway of the host cell. The *rapI* gene was cloned under the inducible promoter and inserted into the chromosome to enable induction of the MICE system when desired, following the strategy reported earlier (Brophy et al., 2018). The MICE system was used to transfer plasmid pA3D-gfpTi into the eight undomesticated *B. subtilis* strains. The result showed that MICE was able to transfer the plasmid into all eight strains (Figure 2C).

**FIGURE 3:**
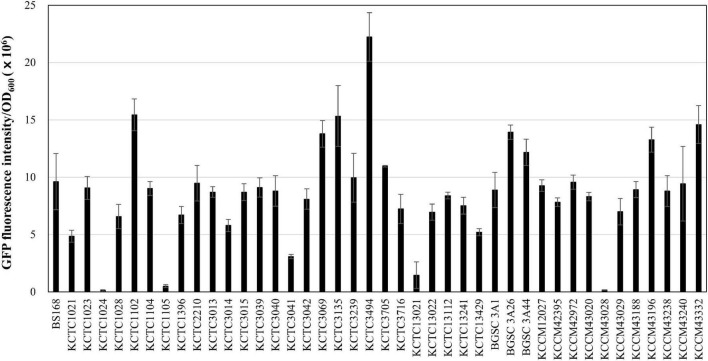
Expression of GFP in 41 undomesticated *B. subtilis* strains. The GFP fluorescence reading was normalized to OD_600_ = 1. The bars display the means of three independent measurements, with the error bars indicating standard deviations.

**FIGURE 4:**
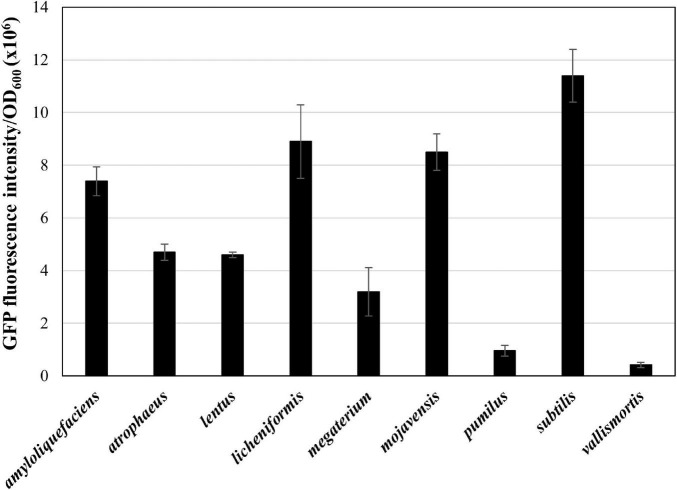
Expression of GFP in QPS *Bacillus* strains. The GFP fluorescence reading was normalized to OD_600_ = 1. The bars display the means of three independent measurements, with the error bars indicating standard deviations.

Next, we applied the MICE system to transfer the *gfp*-carrying plasmid pA3D-gfpTi into 41 undomesticated *B. subtilis* strains listed in Supplementary Table 1, and confirmed that the MICE could transfer the plasmid into all the listed strains. Interestingly, GFP fluorescence intensity varied from strain to strain, indicating that the expression level of heterologous proteins may differ even within the same species (Figure 3). Expansion of the *Bacillus* cell factory beyond *B. subtilis* requires an efficient method of plasmid delivery to various *Bacillus* species. Accordingly, we selected *Bacillus* species from the qualified presumption of safety (QPS) list notified to the European Food Safety Authority (EFSA Panel on Biological Hazards [BIOHAZ], Koutsoumanis et al., 2019) and excluded those that show slow growth or require high temperature and pH for growth. The selected species were *Bacillus amyloliquefaciens*, *Bacillus lentus*, *B. mojavensis*, *B. vallismortis, B. atrophaeus*, *B. licheniformis*, *B. megaterium*, *B. pumilus*, and *B. subtilis* (Supplementary Table 1). Transformation of these strains using the MICE system revealed that the plasmid pA3D-gfpTi was successfully introduced into all of them. Like undomesticated *B. subtilis*, the transformed QPS strains displayed varying expression levels of GFP (Figure 4).

### Cytosine Base Editor-Mediated Multiplex Genome Engineering of Undomesticated *Bacillus* Strains

**FIGURE 5:**
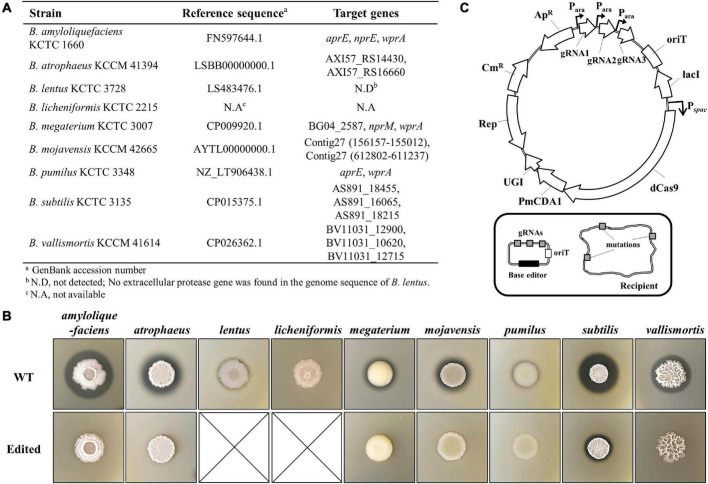
Engineering of the QPS *Bacillus* strains using cytosine base editor. **(A)** Editing targets of the QPS *Bacillus* strains. In the case of *B. lentus*, no extracellular protease genes were found in the genome sequence. *B. licheniformis* used in this study did not have a genome sequence in the NCBI database. **(B)** The extracellular protease activity of the QPS *Bacillus* strains on the TSA plate containing 1% skim milk. *B. lentus* and *B. licheniformis* showed no or little extracellular protease activity. The remaining strains showed no or decreased extracellular protease activity in the edited strain compared to the WT. **(C)** Plasmid map for cytosine base editor-mediated multiplex genome editing. The cytosine base editor transferred by the MICE system can simultaneously edit the genome of the recipient.

For the engineering of the QPS strains, we chose AprE-, NprE-, and WprA-like extracellular proteases as engineering targets as they can break down secreted heterologous proteins or mask the activity of heterologous proteases (Figure 5A). *B. lentus* showed weak extracellular protease activity on a TSA plate containing 1% skim milk (Figure 5B), but the corresponding gene could not be found in the genome sequence deposited in the National Center for Biotechnology Information (NCBI) database. *B. licheniformis* used in this study has neither a genome sequence in the NCBI database nor extracellular protease activity (Figure 5B). Thus, except *B. lentus* and *B. licheniformis*, the other seven strains were engineered to inactivate the target extracellular protease genes. For multiplex genome engineering of these strains, we used a CBE system consisting of a dCas9, a *Petromyzon marinus* cytidine deaminase (PmCDA1), and a uracil DNA glycosylase inhibitor (UGI) (Figure 5C), which can generate stop codons within the multiple target genes simultaneously with high efficiency (Kim et al., 2021). The gRNAs designed to create a stop codon within each protease gene are presented in Supplementary Figure 1. The CBE plasmids containing multiple gRNAs targeting major extracellular proteases were constructed (Supplementary Table 3) and introduced into the QPS strains using the MICE system. Except *B. vallismortis*, the plasmids were successfully transferred to all QPS *Bacillus* strains tested. Four transconjugants of each QPS strains, except *B. megaterium*, were randomly selected, and the introduction of stop codons into target genes was analyzed by sequencing. We confirmed that all target genes were edited simultaneously with 100% efficiency. For *B. megaterium*, only two transconjugants were obtained. Sequencing analysis revealed that all three target genes were edited in one transconjugant. In the other transconjugant, two out of three target genes were edited. Transconjugants for *B. vallismortis* were not obtained through the MICE system. We assumed that the P*_*spac*_* promoter used for the CBE expression was not tightly controlled, and the leaky expression of CBE could have a toxic effect on the growth of *B. vallismortis* cells. Therefore, the promoter P*_*spac*_* was replaced by P*_*xylA*_*, which is known as a more tightly controlled promoter than P*_*spac*_* in *B. subtilis* (Bhavsar et al., 2001). In addition, the MICE donor was further mutated in the *sigK* gene to construct a sporulation-negative donor BS5918, which can aid in the screening of transconjugants. Using the new plasmid and donor, three *B. vallismortis* transconjugants containing the CBE plasmid were obtained. Sequencing analysis confirmed that all target genes were edited in the three transconjugants. The edited QPS strains showed no or significantly decreased protease activities on TSA plate containing 1% skim milk compared to wild-type (WT) strains (Figure 5B). We reconfirmed the decreased protease activities of the edited QPS strains through the protease assay using azocasein as a substrate (Supplementary Figure 2).

### Heterologous Expression of a Nattokinase in Engineered Qualified Presumption of Safety Strains

**FIGURE 6:**
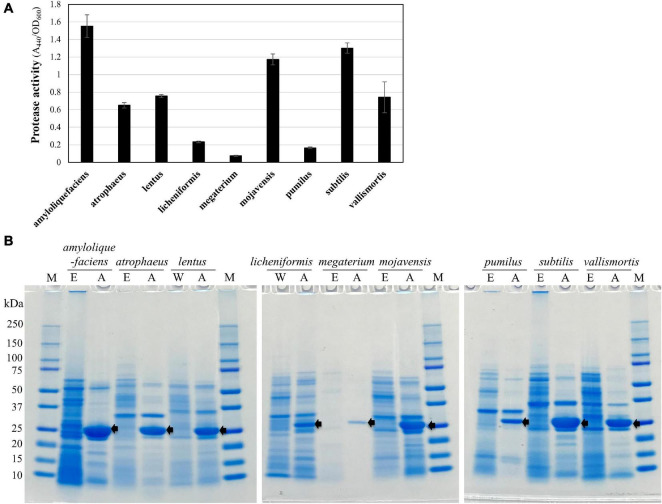
Expression of a nattokinase gene (*aprN*) in edited QPS *Bacillus* strains. **(A)** Protease assay using azocasein as a substrate to determine protease activity of nattokinase-expressing QPS strains. The bars display the means of three independent experiments, with the error bars indicating standard deviations. **(B)** SDS-PAGE analysis of the total protein extracted from the 24-h culture supernatants of the QPS *Bacillus* strains. Protein molecular mass markers are indicated on the “M” lanes. The lanes marked with “W,” “E,” and “A” represent wild type, edited, and *aprN*-expressing strains, respectively. The size of AprN mature protease was 27.7 kDa, indicated by arrows.

The engineered QPS strains were used as heterologous expression hosts for the expression of aprN encoding the fibrinolytic enzyme nattokinase. The plasmid pm3D-aprNTi for expressing *aprN* was constructed (Supplementary Table 3) and introduced into engineered QPS *Bacillus* strains and wild type of *B. lentus* and *B. licheniformis* through the MICE system. The results showed that all QPS strains were successfully transformed with the plasmid, and produced nattokinase. The protease activity of the nattokinase varied among QPS strains, and high activity was observed in *B. amyloliquefaciens*, *B. subtilis*, and *B. mojavensis* (Figure 6A). Nattokinase productivity in the QPS strains was confirmed through SDS-PAGE analysis (Figure 6B). Taken together, we showed through this study that various undomesticated *Bacillus* strains can be engineered and used as cell factories.

## Discussion

The MICE system for highly efficient plasmid delivery into undomesticated *Bacillus* strains was modified from the previously reported mini-ICE*Bs1* system (Brophy et al., 2018) since the previous system cannot be used for marker-free genome engineering of undomesticated *Bacillus* strains. Although the mini-ICE*Bs1* system exhibits broad-host-range characteristics, the ICE*Bs1* inhibits host cell acquisition of a second copy, which may reduce the efficiency of conjugation between the ICE*Bs1*-harboring strains. To date, three inhibition mechanisms have been reported; inhibition of PhrI-mediated cell signaling, ImmR repressor-mediated immunity, and exclusion mechanism based on specificity between donor’s conjugation protein ConG and recipient’s exclusion protein YddJ (Avello et al., 2019). Both mini-ICE*Bs1* and the MICE systems circumvent the first mechanism by overexpressing the regulator RapI. The ImmR-mediated inhibitory mechanism, which suppresses integration of incoming ICE*Bs1* element into the recipient chromosome, is circumvented in MICE system as it only transfers a plasmid and need not to be integrated into the recipient chromosome. The ConG-YddJ-mediated exclusion mechanism remains to be overcome in both mini-ICE*Bs1* and the MICE systems. Nevertheless, our MICE-mediated conjugation resulted high efficiency in various *B. subtilis* strains, similar to that of the mini-ICE*Bs1* system reported earlier (Brophy et al., 2018). Thus, MICE-mediated conjugation efficiency is sufficiently high to transfer plasmids into ICE*Bs1*-containing recipient strains, although the ConG-YddJ-mediated exclusion mechanism may reduce the efficiency.

This study showed that natural competence could not transfer plasmids to undomesticated *B. subtilis* strains tested (Figure 1). The result may be due to a lack of natural competence-related genes or the negative interplay between biofilm formation and competence (She et al., 2020). Some strains may have competence inhibitor genes (Konkol et al., 2013). This study also showed that the plasmid was introduced into four out of eight undomesticated *B. subtilis* strains *via* electroporation (Figure 1). The method showed inconsistent results, indicating that optimization of the electroporation conditions for each strain is required (Figure 1). Several conjugation methods, such as RP4 transfer system (Simon et al., 1983), pLS20 plasmid system (Koehler and Thorne, 1987), and ICE*Bs1* (Johnson and Grossman, 2015) have been reported in *Bacillus* strains. In this study, the EBC using the RP4 transfer system, and the BBC using the pLS20 plasmid showed consistent results and broader range compared to the natural competence and electroporation methods, but some undomesticated *Bacillus* strains still remained untransformed (Figure 1). The newly developed MICE system in this study showed a wider host range than other systems. It transformed not only undomesticated *B. subtilis* but also QPS *Bacillus* species (Figures 3, 4). The reason for the wide host range can possibly be the increased availability of conjugation machinery due to the overexpression a regulator RapI that induces the expression of the MICE genes. The applicability of MICE system in a broad-host-range shall guide the future studies on the marker-free genome editing of other microorganisms beyond *Bacillus* species.

CRISPR-Cas9-mediated genome engineering has been well established with high efficiency in *B. subtilis* (Altenbuchner, 2016; Westbrook et al., 2016; So et al., 2017). Since the CRISPR-Cas9 system requires a gRNA and two flanking DNA fragments for homologous recombination, plasmids for simultaneous multi-genome editing are not simple in construction and large in size, which can reduce transformation efficiency. Moreover, simultaneous multiple editing with Cas9 produces multiple fragmentation of the chromosome, which can result in very low editing efficiency. There is a recent report of simultaneous editing of three targets for point mutations using Cas9 nickase in *B. subtilis* (Liu et al., 2019). It showed considerable mutation efficiency, but still needed donor DNA fragments. A recently developed CBE system enables precise base editing in yeast, mammalian cells, and bacteria without double-stranded break (Komor et al., 2017). The independence of donor DNA fragments can simplify the cloning process and reduce the size of the plasmid. A CBE system for *B. subtilis* has been reported as a powerful tool for multiplex genome editing due to its characteristics of simplicity, high efficiency, high specificity, and low genomic damage. It has generated off-target mutations in about 8.5 single nucleotide variants in CBE-edited strains, but most off-target mutations can be silent or harmless (Kim et al., 2021). Even if there are detrimental mutations that affect major cellular physiology, they can be filtered out by subsequent screening of growth and physiological characteristics. In this study, we showed that the CBE system can simultaneously edit multiple genes in various *Bacillus* species as well as *B. subtilis*, indicating that various undomesticated *Bacillus* strains isolated from environments could be engineered for cell factories. In the future, various other CRISPR tools could be transferred into undomesticated *Bacillus* strains using the MICE system for cell factory engineering.

*Bacillus* strains have been isolated from various environments, such as soil, plant, food, marine sediments, and the human gut with a wide range of pH, temperatures, and salts. They produce versatile enzymes and secondary metabolites and are notable resources for the discovery of novel antibiotics (Aleti et al., 2015; Zhao and Kuipers, 2016; Grubbs et al., 2017). In addition to traditional fields, such as industry and agriculture, *Bacillus* strains are considered to have great potential in medical applications. To realize their potential, *Bacillus* research should be shifted from laboratory strains to undomesticated ones. Therefore, the systems for engineering undomesticated strains developed in this study could allow for breakthroughs in research for the utilization of untapped *Bacillus* resources.

## Data Availability Statement

The original contributions presented in the study are included in the article/Supplementary Material, further inquiries can be directed to the corresponding author/s.

## Author Contributions

D-EJ, MSK, and S-KC designed the experiments and wrote the manuscript. D-EJ, MSK, and H-RK realized all experiments. All authors reviewed the manuscript and contributed to the article and approved the submitted version.

## Conflict of Interest

The authors declare that the research was conducted in the absence of any commercial or financial relationships that could be construed as a potential conflict of interest.

## Publisher’s Note

All claims expressed in this article are solely those of the authors and do not necessarily represent those of their affiliated organizations, or those of the publisher, the editors and the reviewers. Any product that may be evaluated in this article, or claim that may be made by its manufacturer, is not guaranteed or endorsed by the publisher.
