# Supplementary material for: Cell Factory Engineering of Undomesticated Bacillus Strains Using a Modified Integrative and Conjugative Element for Efficient Plasmid Delivery
Source: Front Microbiol. 2022 Apr 26;13:802040. doi: 10.3389/fmicb.2022.802040 (PMC9086855; doi:10.3389/fmicb.2022.802040)
Supplement: Supplementary file 1 [file Data_Sheet_1.docx]

**Supplementary** **Materials**

The following Supplementary Materials are available for this article:

**Supplementary Figure S1.** The chromosomal gRNA target sites for introducing a stop-codon into each protease gene of QPS strains.

**Supplementary Figure S2.** The protease assay using azocasein as a substrate with culture supernatants of QPS *Bacillus* strains.

**Supplementary Table S1.** Recipient strains used in this study

**Supplementary Table S2.** Donor strains used in this study

**Supplementary Table S3.** Plasmids used in this study

**Supplementary Table S4.** Primers used in this study

**Supplementary References**


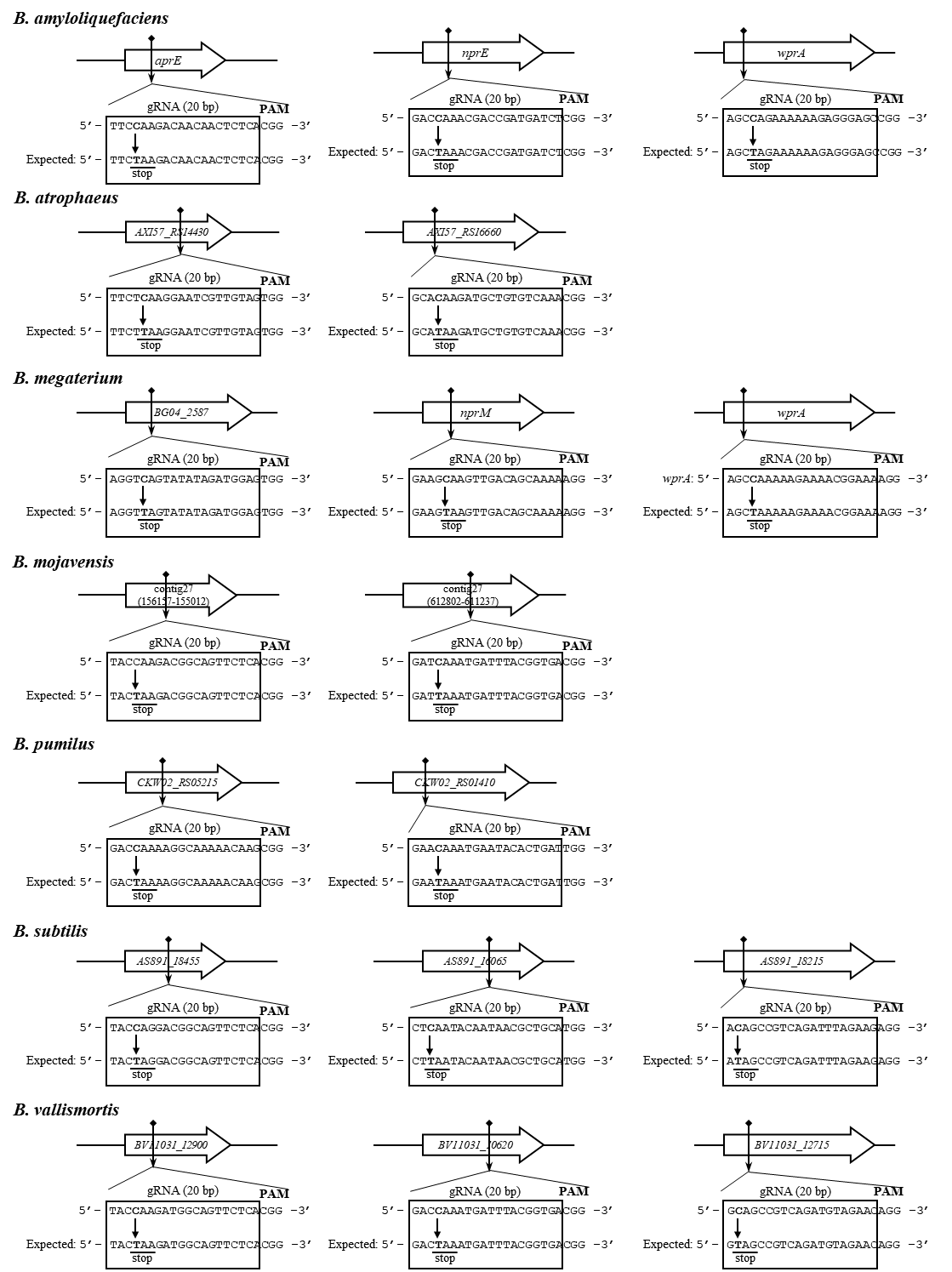


**Supplementary Figure S1.** Chromosomal gRNA target sites for introducing a stop-codon into each protease gene of QPS strains. The gRNA-binding sites are indicated by the black boxes including the target bases (bold) and the expected codons (underline).

**
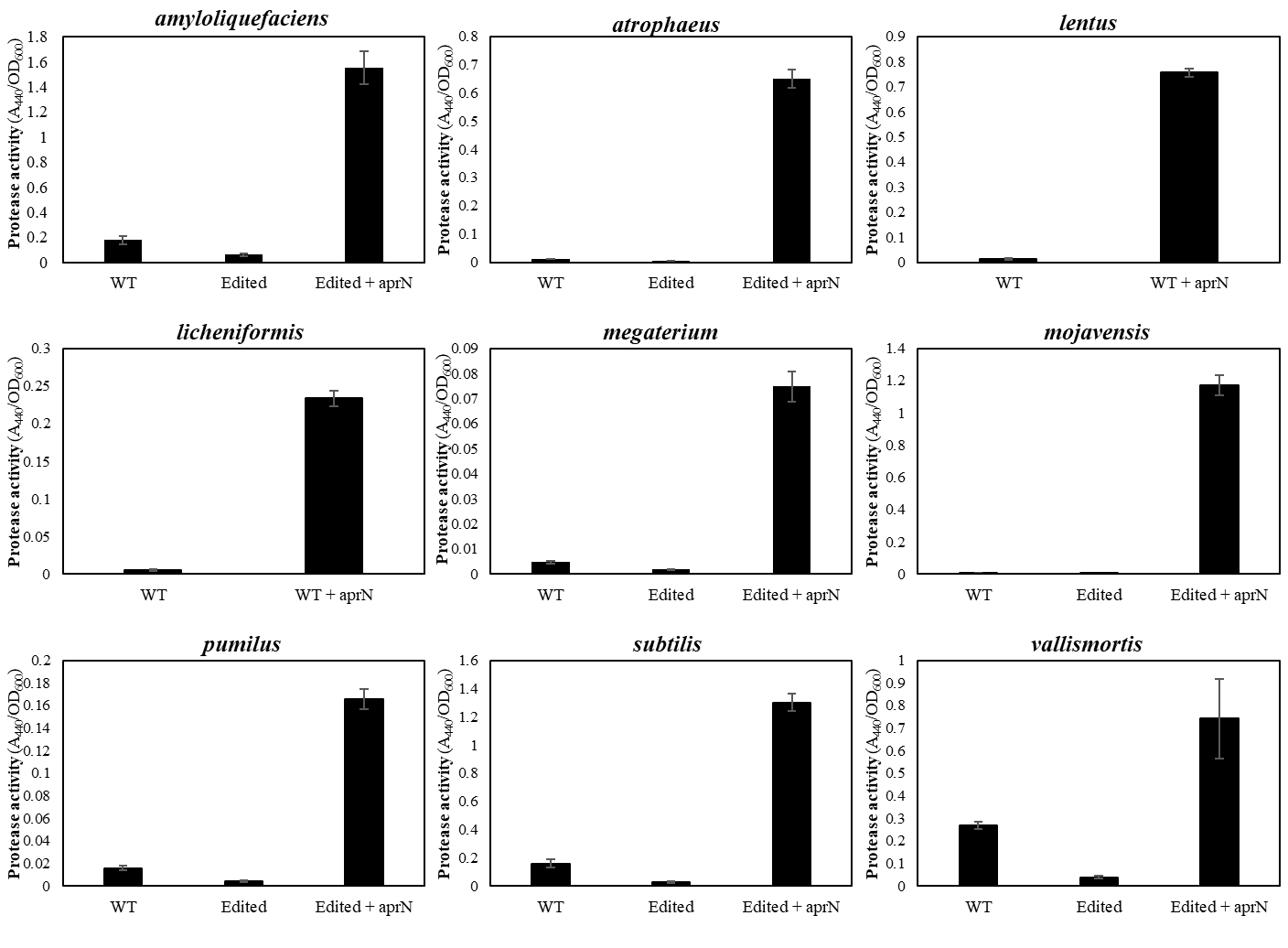
**

**Supplementary Figure S2.** Protease assay using azocasein as a substrate with culture supernatants of QPS *Bacillus* strains. The absorbance reading (440nm) was normalized to OD_600_=1. The bars display the means of three independent experiments, with the error bars indicating standard deviations.

**Supplementary Table S1.** Recipient strains used in this study

| **Strain** | **Source^b^** | **Growth conditions** |
| --- | --- | --- |
| *Bacillus spizizenii^a^* | KCTC 1021 | LB medium/ 37℃ |
| *Bacillus subtilis* | KCTC 1023 | LB medium/ 37℃ |
| *Bacillus subtilis* | KCTC 1024 | LB medium/ 37℃ |
| *Bacillus subtilis* | KCTC 1028 | LB medium/ 37℃ |
| *Bacillus subtilis* | KCTC 1102 | LB medium/ 37℃ |
| *Bacillus subtilis* | KCTC 1104 | LB medium/ 37℃ |
| *Bacillus subtilis* | KCTC 1105 | LB medium/ 37℃ |
| *Bacillus subtilis* | KCTC 1396 | LB medium/ 37℃ |
| *Bacillus subtilis* | KCTC 2210 | LB medium/ 37℃ |
| *Bacillus subtilis* | KCTC 3013 | LB medium/ 37℃ |
| *Bacillus subtilis* | KCTC 3014 | LB medium/ 37℃ |
| *Bacillus subtilis* | KCTC 3015 | LB medium/ 37℃ |
| *Bacillus subtilis* | KCTC 3039 | LB medium/ 37℃ |
| *Bacillus subtilis* | KCTC 3040 | LB medium/ 37℃ |
| *Bacillus subtilis* | KCTC 3041 | LB medium/ 37℃ |
| *Bacillus subtilis* | KCTC 3042 | LB medium/ 37℃ |
| *Bacillus subtilis* | KCTC 3069 | LB medium/ 37℃ |
| *Bacillus subtilis^c^* | KCTC 3135 | LB medium/ 37℃ |
| *Bacillus subtilis* | KCTC 3239 | LB medium/ 37℃ |
| *Bacillus subtilis* | KCTC 3494 | LB medium/ 37℃ |
| *Bacillus spizizenii^a^* | KCTC 3705 | LB medium/ 37℃ |
| *Bacillus* *subtilis* | KCTC 3716 | LB medium/ 37℃ |
| *Bacillus subtilis* | KCTC 13021 | LB medium/ 37℃ |
| *Bacillus subtilis* | KCTC 13022 | LB medium/ 37℃ |
| *Bacillus subtilis* | KCTC 13112 | LB medium/ 37℃ |
| *Bacillus subtilis* | KCTC 13241 | LB medium/ 37℃ |
| *Bacillus inaquosorum^a^* | KCTC 13429 | LB medium/ 37℃ |
| *Bacillus subtilis* Marburg NCIB3610 | BGSC 3A1 | LB medium/ 37℃ |
| *Bacillus subtilis* AUSI98 | BGSC 3A26 | LB medium/ 37℃ |
| *Bacillus subtilis* RO-NN-1 | BGSC 3A44 | LB medium/ 37℃ |
| *Bacillus subtilis* natto | KCCM 12027 | LB medium/ 37℃ |
| *Bacillus subtilis* | KCCM 42395 | LB medium/ 37℃ |
| *Bacillus spizizenii^a^* | KCCM 42972 | LB medium/ 37℃ |
| *Bacillus subtilis* 21-B-4 | KCCM 43020 | LB medium/ 37℃ |
| *Bacillus subtilis* C17Y16 | KCCM 43028 | LB medium/ 37℃ |
| *Bacillus subtilis* C11Y16 | KCCM 43029 | LB medium/ 37℃ |
| *Bacillus subtilis* SB-1 | KCCM 43188 | LB medium/ 37℃ |
| *Bacillus subtilis* FBL-1 | KCCM 43196 | LB medium/ 37℃ |
| *Bacillus subtilis* BS38 | KCCM 43238 | LB medium/ 37℃ |
| *Bacillus subtilis* BS16045 | KCCM 43240 | LB medium/ 37℃ |
| *Bacillus subtilis* MBEL3395 | KCCM 43332 | LB medium/ 37℃ |
| *Bacillus amyloliquefaciens^c^* | KCTC 1660 | LB medium/ 37℃ |
| *Bacillus atrophaeus^c^* | KCCM 41394 | LB medium/ 30℃ |
| *Bacillus lentus^c^* | KCTC 3728 | LB medium/ 30℃ |
| *Bacillus licheniformis^c^* | KCTC 2215 | LB medium/ 37℃ |
| *Bacillus megaterium^c^* | KCTC 3007 | TSB medium/ 30℃ |
| *Bacillus mojavensis^c^* | KCCM 42665 | LB medium/ 30℃ |
| *Bacillus pumilus^c^* | KCTC 3348 | TSB medium/ 30℃ |
| *Bacillus vallismortis^c^* | KCCM 41614 | LB medium/ 30℃ |

^a^In this study, *Bacillus spizizenii* and *Bacillus inaquosorum* were used as subspecies of *B. subtilis*, but were elevated to independent species status in 2020 (Dunlap et al., 2020).

^b^Strains were obtained from Korean Collection for Type Cultures (KCTC), *Bacillus* Genetic Stock Center (BGSC), or Korean Culture Center of Microorganisms (KCCM).

^c^QPS strains used in this study

**Supplementary Table S2.** Donor strains used in this study

| **Strain** | **Genotype** | **Reference** |
| --- | --- | --- |
| **For EBC** |  |  |
| *Escherichia coli* S17-1 | *recA* *pro* *hsdR* RP4-2-Tc::Mu-Km::Tn7 integrated into the chromosome | KCTC 2432 |
| **For BBC** |  |  |
| *Bacillus subtilis* natto | *bio*^-^ with plasmids pLS19 and pLS20 | KCCM 12512 |
| BS5879 | *B. subtilis* natto KCCM 12512 with plasmid pBC16 | This study |
| *B. subtilis* BSK1 | B. subtilis 168 △BsuM | (Choi et al., 2009) |
| BS5417 | *B. subtilis* 168 *thrC*::P*_xyl_-comK* | (Jeong et al., 2015) |
| BS5418 | BSK1 *thrC*::P*_xyl_-comK* | This study |
| BS5887 | BS5418 with plasmid pLS20 | This study |
| BS5892 | BS5887 △*alrA* | This study |
| **For MICE** |  |  |
| *Bacillus subtilis* 168 | *trpC2* | Laboratory stock |
| BS5889 | *B. subtilis* 168 △*rapI*-*attR*::*hyg*^R^ | This study |
| BS5890 | BS5889 △*alrA*::Sp^R^-P*_xylA_*-*rapI* | This study |
| BS5899 | BS5890 △*oriT*_ICE_ | This study |
| BS5918 | BS5899 △*sigK* | This study |

**Supplementary Table S3.** Plasmids used in this study

| **Plasmid** | | **Description** | **Reference** |
| --- | --- | --- | --- |
| pBC16 | Template for amplifying tetracycline resistance gene | | (Palva et al., 1990) |
| pAX01 | Template for amplifying promoter P*_xylA_* | | (Härtl et al., 2001) |
| pDG1661 | Integration vector for *Bacillus subtilis* | | (Guérout-Fleury et al., 1996) |
| pDG1728 | Template for amplifying spectinomycin resistance gene | | (Guérout-Fleury et al., 1996) |
| pGEM-T Easy | Plasmid for cloning of PCR products, Amp^R^ | | Promega |
| pGEM-*rapI* | pGEM-T Easy derivative for generating P*_xylA_*-*rapI* insertion at *alrA* of *Bacillus* donor | | This study |
| pAgR | Plasmid pAD123 derivative containing synthetic sgRNA  Module | | (So et al., 2017) |
| pMGold-sCBE4 | The modified pAgR; Golden-Gate assembly site for sgRNA cloning and cytosine base editor (CBE4) expressing under P*_spac_* | | (Kim et al., 2021) |
| pMGoldi-sCBE4 | pMGold-sCBE4 derivative containing *oriT*_ICE_ (for MICE) instead of *oriT* (for EBC) | | This study |
| pMGoldi-xCBE4 | pMGoldi-sCBE4 derivative replacing P*_spac_* with P*_xylA_* | | This study |
| pUC18/pUC19 | Cloning vector | | Laboratory stock |
| pAD123 | *E. coli-Bacillus* shuttle vector | | (Dunn and Handelsman, 1999) |
| pA3D-gfpTe | pAD123 derivative containing P*_cry3Aa_*-gfp cassette and *oriT*_e_ (RP4) | | This study |
| pA3D-gfpT20 | pAD123 derivative containing P*_cry3Aa_*-gfp cassette and *oriT*_20_ (pLS20) | | This study |
| pA3D-gfpTi | pAD123 derivative containing P*_cry3Aa_*-gfp cassette and *oriT*_ICE_ (BS168) | | This study |
| pACas9T | pAD123 derivative with P*_spac_*-SpCas9 and sgRNA cassette | | This study |
| pACas9T-gTc | pACas9T derivative with gRNA targeting pBC16-encoded *tet* | | This study |
| pACas9T-dalrA | pACas9T derivative with gRNA targeting *alrA* and 1 kb donor DNA | | This study |
| pUC-rapI::hyg | pUC18 derivative, generating hyg^R^ insertion at *rapI-attR* | | This study |
| pACas9T-dnicK | pACas9T derivative with gRNA targeting *nick* and 1 kb donor DNA | | This study |
| pACas9T-dsigK | pACas9T derivative with gRNA targeting *sigK* and 1 kb donor DNA | | This study |
| pMsCBE4i-amANW | pMGold-*sCBE4* with sgRNAs: amy-Ag, amy-Ng, and amy-Wg of *B. amylolquefaciens* | | This study |
| pMsCBE4i-atAN | pMGold-*sCBE4* with sgRNAs: atr-Ag and atr-Ng of *B. atrophaeus* | | This study |
| pMsCBE4i-meANW | pMGold-*sCBE4* with sgRNAs: meg-Ag, meg-Ng, and meg-Wg of *B. megaterium* | | This study |
| pMsCBE4i-moAN | pMGold-*sCBE4* with sgRNAs: moj-Ag and moj-Ng of *B. mojavensis* | | This study |
| pMsCBE4i-puAW | pMGold-*sCBE4* with sgRNAs: pum-Ag and pum-Wg of *B. pumilus* | | This study |
| pMsCBE4i-suANW | pMGold-*sCBE4* with sgRNAs: *aprE*-stop, *nprE*-stop, and *wprA*-stop of *B. subtilis* | | This study |
| pMsCBE4i-vaANW | pMGold-*sCBE4* with sgRNAs: val-Ag, val-Ng, and val-Wg of *B. vallismortis* | | This study |
| pMxCBE4i-vaANW | pMGold-*xCBE4* with sgRNAs: val-Ag, val-Ng, and val-Wg of *B. vallismortis* | | This study |
| pm3D-aprNTi | pMGold-sCBE4 derivative containing P*_cry3Aa_*-aprN cassette and *oriT*_ICE_ (BS168) | | This study |
| pHCas9 | pHT01 derivative plasmid containing P*_grac_*-SpCas9 | | (So et al., 2017) |
| pHPspac | IPTG-inducible vector | | (Kim et al., 2014) |
| pHCas9spac | pHPspac derivative containing P*_spac_*-SpCas9 | | This study |

**Supplementary Table S4.** Primers used in this study

| **Oligonucleotide** | **Sequence (5’ to 3’)** | **Purpose** |
| --- | --- | --- |
| Tc-gF1 | ATTG**TGATAGCCCATTATATTCAT** | Oligonucleotides for cloning of synthetic gRNA sequence used in this study |
| Tc-gR1 | AAAC**ATGAATATAATGGGCTATCA** |  |
| alrA-gF | ATTG**AACCCTCGTTTAAAGTGCAA** |  |
| alrA-gR | AAAC**TTGCACTTTAAACGAGGGTT** |  |
| nicK-gF2 | ATTG**ACAACCCCCCCACGCTAACA** |  |
| nicK-gR2 | AAAC**TGTTAGCGTGGGGGGGTTGT** |  |
| sigK-gF1 | ATTG**GCAGAGGACTTAATCTCCAT** |  |
| sigK-gR1 | AAAC**ATGGAGATTAAGTCCTCTGC** |  |
| amy-AgF | ATTG**TTCCAAGACAACAACTCTCA** |  |
| amy-AgR | AAAC**TGAGAGTTGTTGTCTTGGAA** |  |
| amy-NgF | ATTG**GACCAAACGACCGATGATCT** |  |
| amy-NgR | AAA**CAGATCATCGGTCGTTTGGTC** |  |
| amy-WgF | ATTG**AGCCAGAAAAAAGAGGGAGC** |  |
| amy-WgR | AAAC**GCTCCCTCTTTTTTCTGGCT** |  |
| atr-AgF | ATTG**TTCTCAAGGAATCGTTGTAG** |  |
| atr-AgR | AAAC**CTACAACGATTCCTTGAGAA** |  |
| atr-NgF | ATTG**GCACAAGATGCTGTGTCAAA** |  |
| atr-NgR | AAAC**TTTGACACAGCATCTTGTGC** |  |
| meg-AgF | ATTG**AGGTCAGTATATAGATGGAG** |  |
| meg-AgR | AAAC**CTCCATCTATATACTGACCT** |  |
| meg-NgF | ATTG**GAAGCAAGTTGACAGCAAAA** |  |
| meg-NgR | AAAC**TTTTGCTGTCAACTTGCTTC** |  |
| meg-WgF | ATTG**AGCCAAAAAGAAAACGGAAA** |  |
| meg-WgR | AAAC**TTTCCGTTTTCTTTTTGGCT** |  |
| moj-AgF | ATTG**TACCAAGACGGCAGTTCTCA** |  |
| moj-AgR | AAAC**TGAGAACTGCCGTCTTGGTA** |  |
| moj-NgF | ATTG**GATCAAATGATTTACGGTGA** |  |
| moj-NgR | AAAC**TCACCGTAAATCATTTGATC** |  |
| pum-AgF | ATTG**GACCAAAAGGCAAAAACAAG** |  |
| pum-AgR | AAAC**CTTGTTTTTGCCTTTTGGTC** |  |
| pum-WgF | ATTG**GAACAAATGAATACACTGAT** |  |
| pum-WgR | AAAC**ATCAGTGTATTCATTTGTTC** |  |
| *aprE*-stop-F | ATTG**TACCAGGACGGCAGTTCTCA** |  |
| *aprE*-stop-B | AAAC**TGAGAACTGCCGTCCTGGTA** |  |
| *nprE*-stop-F | ATTG**CTCAATACAATAACGCTGCA** |  |
| *nprE*-stop-B | AAAC**TGCAGCGTTATTGTATTGAG** |  |
| *wprA*-stop-F | ATTG**ACAGCCGTCAGATTTAGAAG** |  |
| *wprA*-stop-B | AAAC**CTTCTAAATCTGACGGCTGT** |  |
| val-AgF | ATTG**TACCAAGATGGCAGTTCTCA** |  |
| val-AgR | AAAC**TGAGAACTGCCATCTTGGTA** |  |
| val-NgF | ATTG**GACCAAATGATTTACGGTGA** |  |
| val-NgR | AAAC**TCACCGTAAATCATTTGGTC** |  |
| val-WgF | ATTG**GCAGCCGTCAGATGTAGAAC** |  |
| val-WgR | AAAC**GTTCTACATCTGACGGCTGC** |  |
| **Primer** | **Sequence (5’ to 3’)** | **Purpose** |
| 3Aag-F2 | AGCTGTCAAACATGAGAATTCGAGCTCTCAGCAGTAGAAG | Primers for construction of the pD3D-gfp |
| stabR2 | TTTTCTTCCTCCCTTTCTTA |  |
| gfp-F1 | TAAGAAAGGGAGGAAGAAAAATGAGTAAAGGAGAAGAACTT |  |
| gfp-R1 | TTTCGCTCGGGAAGACGTACGTTATTTGTATAGTTCATCCA |  |
| oriTe-AatF | GAAAAGTGCCACCTGACGTCAAAGAATTCAAAGGATCCAAAACTAGTTCTTGACGAGTTCTTCTGAGCG | Primers for construction of the pA3D-gfpTe |
| oriTe-SacR | CTTCTACTGCTGAGAGCTCTTTGTCGACTTTGCATGCTTTTCTAGAATCGTCTCTCGCCTGTCCCCTC |  |
| oriT20-BamF | ACGTCAAAGAATTCAAAGGATCCAAAGAGCAATCTCGTCATCGAAGACTAA | Primers for construction of the pA3D-gfpT20 |
| oriT20-XbaR | CGACTTTGCATGCTTTTCTAGATTGTTAACGCTCCTTTTCATC |  |
| oriTICE-BamF | ACGTCAAAGAATTCAAAGGATCCCAGGCGGGGTATTTTCTCGTTTTAGG | Primers for construction of the pA3D-gfpTi |
| oriTICE-XbaR | CGACTTTGCATGCTTTTCTAGACTTTTGGGCTTTCTTTAACAGTTCT |  |
| P3D-sacF | AGCATGCAAAGTCGACAAAGAGCTCTCAGCAGTAGAAGTTTTGACCAAAATTAAAAAAATACCCA | Primers for construction of the pm3D-aprNTi |
| aprE-F2 | TAAGAAAGGGAGGAAGAAAAATGAGAAGCAAAAAATTGTG |  |
| aprE-BWR | TTTCGCTCGGGAAGACGTACGTTATTGTGCAGCTGCTTGTACGTTGATTAA |  |
| alrA-SpeF | CCATTTGTATTGTGTTGAACTAGTCAGGGTTTATTTAGAAAATCCGAA | Primers for donor template of the pACas9T-dalrA |
| alrA-FR | TACGACACTTCCTAGCTTTTATTCA |  |
| alrA-BF | TTGAATAAAAGCTAGGAAGTGTCGTATAAAACAGCTTTGCTTGAAGAGTG |  |
| alrA-XmaR | GAAGGCGATAGACGTCTTTCCCGGGTCAAATTCGCTTACCTCCCAATAAA |  |
| yddJ-H3F | TAAAACGACGGCCAGTGCCAAGCTTGATGTAATTAGCCAATATAAAGAG | Primers for construction of the pUC-rapI::hyg |
| yddJ-hygR | TCGTCCACAGTTCGCGGCTAGCTAAGCAGCATAACTCACTTTTCAAG |  |
| hyg-R | TTAGCTAGCCGCGAACTGTGGACGAGAACT |  |
| hyg-F | ATTCTCGAGGCTTTAATGCGGTAGTTTATCA |  |
| yddN-hygF | ACTACCGCATTAAAGCCTCGAGAATCGTTCATTGTAAAAGAAACGAACCATGTCCTATT |  |
| yddN-EcoR | CAGCTATGACCATGATTACGAATTCGCGTAGGTTCAGGGGGTATTATGTTGC |  |
| alr_front_F | TATACAATGTGGAAATCTGGC | Primers for construction of the pGEM-rapI |
| alr_front_R | TACGACACTTCCTAGCTTTTATTC |  |
| alr_back_F | GCAGCAGTGATTTTAAGTAGGACATATTTGCAGGTTGCTC |  |
| alr_back_R | GTTATTCCCCCAATGAAACG |  |
| spcF | GAATAAAAGCTAGGAAGTGTCGTACGATTTTCGTTCGTGAATAC |  |
| spcR | TATGCAAGGGTTTATTGTTTTC |  |
| xyl-F | GAAAACAATAAACCCTTGCATAAGATCTTGATTAATTAATTCAGAAC |  |
| xyl-R | TTGTCATTTCCCCCTTTGATTTTTAG |  |
| rapI-F | CTAAAAATCAAAGGGGGAAATGACAATTGCGGGGTGTTTTCTTAGA |  |
| rapI-R | CTACTTAAAATCACTGCTGC |  |
| nicK-SpeF | CCATTTGTATTGTGTTGAACTAGTTGATGCAGTAGCTGAGAATGGAGTCC | Primers for donor template of the pACas9T-dnicK |
| nicK-FR | ACTACTCCTCTGTTAGCGTGTGGTGGTTGTTTTAGCTCATCCATTCGCTTCGCTCACTCC |  |
| nicK-BF | CACCACACGCTAACAGAGGAGTAGTAATCGTAAAAGAAAAAAATGAAGCCGTTGAAAG |  |
| nicK-XmaR | GAAGGCGATAGACGTCTTTCCCGGGCATACGTTTAATCGCATCACCTGTGTTT |  |
| sigK-SpeF | CCATTTGTATTGTGTTGAACTAGTGGCATCGTTGTACGGGACGAAGGCAC | Primers for donor template of the pACas9T-dsigK |
| sigK-R | CATCGTCACCTCCACAAAAGTA |  |
| sigK-F | ACATACTTTTGTGGAGGTGACGATGAGTTAAGTTATCTGCACCGATTGA |  |
| sigK-XmaR | GAAGGCGATAGACGTCTTTCCCGGGAAAGTATGGCACAGGCAGGTT |  |
| ICE-OriT-F | GTGCTTTTTTTGGAAAGCTTCAGGCGGGGTATTTTCTCGT | Primers for construction of the pMGoldi-xCBE4 |
| ICE-OriT-R | GTGAGTTAAGGCCTGAGCTCCTTTTGGGCTTTCTTTAACAGTTC |  |
| xylR-xyl-F | GAGACGATGCCAAAGAGCTCAGATCTTGATTAATTAATTCAGAACGCTCGG |  |
| xylR-xyl-R | AGACTAGTTTTTGGCCGGCCTTAAGTGAACAAGTTTATCCATC |  |
| Cas9F | AAGGAGGTGAGGATCCAAAGAGGAGAAAGGATCTATGGATAA | Primers for construction of the pACas9T |
| Cas9R | CTTAGATCTCCCGGGTCCCTAGGTATAAACGCAGA |  |
| pADallF | GGTGACTAACTCGAGAAATGTACAAATAGATCTAAAGCCGGCAATATGCATCTGTCAGACCAAGTTTACTC |  |
| pADallR | GGTTTCTTAGACGTCTTTCCCGGGTTTGTTTAAACATTTCTAGATTTACTAGTTCAACACAATACAAATGGGTTAG |  |
| oriTF | CCCGGGAAAGACGTCTATCGCCTTCTTGACGAGTT |  |
| oriTR | AAGAAAAACCACCCTGGCGCCCAATACGCAAACCGCCTCTCCCC |  |
| gRNAF | GGTGACTAACTCGAGCCAGCAAGACAGCGATAAAG |  |
| gRNAR | AGATCTATTTGTACAAAAAAAAGCACCGACTCGGTG |  |

Underlined sequences are the restriction enzyme sites.

Bolded sequences represent the 20 bp synthetic gRNA.

**Supplementary References**

Choi, S.-K., Park, S.-Y., Kim, R., Kim, S.-B., Lee, C.-H., Kim, J.F., et al. (2009). Identification of a polymyxin synthetase gene cluster of Paenibacillus polymyxa and heterologous expression of the gene in Bacillus subtilis. *J Bacteriol* 191**,** 3350-3358. doi: 10.1128/JB.01728-08.

Dunlap, C.A., Bowman, M.J., and Zeigler, D.R. (2020). Promotion of Bacillus subtilis subsp. inaquosorum, Bacillus subtilis subsp. spizizenii and Bacillus subtilis subsp. stercoris to species status. *Antonie Leeuwenhoek* 113**,** 1-12. doi: 10.1007/s10482-019-01354-9.

Dunn, A.K., and Handelsman, J. (1999). A vector for promoter trapping in Bacillus cereus. *Gene* 226**,** 297-305. doi: 10.1016/s0378-1119(98)00544-7.

Guérout-Fleury, A.-M., Frandsen, N., and Stragier, P. (1996). Plasmids for ectopic integration in Bacillus subtilis. *Gene* 180**,** 57-61. doi: 10.1016/s0378-1119(96)00404-0.

Härtl, B., Wehrl, W., Wiegert, T., Homuth, G., and Schumann, W. (2001). Development of a new integration site within the Bacillus subtilis chromosome and construction of compatible expression cassettes. *J Bacteriol* 183**,** 2696. doi: 10.1128/JB.183.8.2696-2699.2001.

Jeong, D.-E., Park, S.-H., Pan, J.-G., Kim, E.-J., and Choi, S.-K. (2015). Genome engineering using a synthetic gene circuit in Bacillus subtilis. *Nucleic Acids Res.* 43**,** e42. doi: 10.1093/nar/gku1380.

Kim, H.R., Park, S.Y., Kim, S.B., Jeong, H., Choi, S.K., and Park, S.H. (2014). Inactivation of the phosphoglucomutase gene pgm in Paenibacillus polymyxa leads to overproduction of fusaricidin. *J. Ind. Microbiol. Biotechnol.* 41**,** 1405-1414. doi: 10.1007/s10295-014-1470-z.

Kim, M.S., Kim, H.-R., Jeong, D.-E., and Choi, S.-K. (2021). Cytosine Base Editor-Mediated Multiplex Genome Editing to Accelerate Discovery of Novel Antibiotics in Bacillus subtilis and Paenibacillus polymyxa. *Front. Microbiol.* 12. doi: 10.3389/fmicb.2021.691839.

Palva, A., Vigren, G., Simonen, M., Rintala, H., and Laamanen, P.i. (1990). Nucleotide sequence of the tetracycline resistance gene of pBC16 from Bacillus cereus. *Nucleic Acids Res.* 18**,** 1635. doi: 10.1093/nar/18.6.1635.

So, Y., Park, S.-Y., Park, E.-H., Park, S.-H., Kim, E.-J., Pan, J.-G., et al. (2017). A highly efficient CRISPR-Cas9-mediated large genomic deletion in Bacillus subtilis. *Front. Microbiol.* 8**,** 1167. doi: 10.3389/fmicb.2017.01167.
